# Review of drivers and barriers of water and sanitation policies for urban informal settlements in low-income and middle-income countries

**DOI:** 10.1016/j.jup.2019.100957

**Published:** 2019-10

**Authors:** Sheela S. Sinharoy, Rachel Pittluck, Thomas Clasen

**Affiliations:** Department of Environmental Health, Rollins School of Public Health, Emory University, Atlanta, USA

**Keywords:** Water and sanitation, Informal settlements, Slums

## Abstract

This study examined drivers and barriers of water, sanitation, and hygiene (WASH) policies in urban informal settlements in low and middle-income countries. We conducted a search of peer-reviewed and grey literature published between January 2000 and April 2018. We organized evidence into six domains of drivers and barriers: economic, spatial, social, institutional, political, and informational. Key drivers included donor prioritization and collective action, while key barriers included social exclusion, lack of land or dwelling tenure status, the political economy of decision-making, and insufficient data. Ensuring responsive water and sanitation policies for informal settlements will require inter-disciplinary collaboration and both top-down and bottom-up approaches.

## 1 Introduction

### 1.1 Global burden of unsafe water and sanitation

Water, sanitation, and hygiene (WASH) have long been recognized as important determinants of human health. Inadequate WASH is one of the primary risk factors for diarrheal disease, a leading cause of mortality and disability-adjusted life-years (DALYs) worldwide (GBD Diarrhoeal Diseases Collaborators, 2017). Studies suggest that interventions to provide improved water or sanitation can significantly reduce diarrhea (GBD Diarrhoeal Diseases Collaborators, 2017; Clasen et al., 2015; Wolf et al., 2014). In addition, inadequate water and sanitation have been associated with other adverse outcomes including helminth infections, child under-nutrition, and impaired cognitive development (Dangour et al., 2013; Sclar et al., 2017; Strunz et al., 2014).

Despite widespread awareness of the importance of safe water and sanitation, including through the Millennium Development Goals (MDGs) and the United Nations (UN) International Decade for Action “Water for Life” 2005–2015 (UN, 2015), substantial gaps remain. In 2015, 2.3 billion people still lacked basic sanitation, and 844 million people lacked a basic drinking water service (WHO/UNICEF Joint Monitoring Programme for Water Supply, 2017). The Sustainable Development Goals (SDGs), which succeeded the MDGs, aimed to further increase attention to WASH by seeking to ensure safe drinking water and basic sanitation for all (UN, 2017). Notably, whereas the MDGs only considered toilets, the indicators for the SDGs have been expanded to require “safely managed” water supplies and sanitation.

The SDGs also emphasize equity, both between and within countries (Hutton and Chase, 2016). Within countries, urban populations are far more likely to have sewer connections and piped water supplies (WHO/UNICEF Joint Monitoring Programme for Water Supply, 2017). However, while rural access to drinking water has improved steadily since 1990, urban access has stagnated or improved only marginally (Dos Santos et al., 2017). In some places, the proportion of the urban population with access to basic sanitation or piped water on premises has fallen (WHO/UNICEF Joint Monitoring Programme for Water Supply, 2017; Satterthwaite, 2016). Additionally, within urban areas, disparities exist in access between the urban rich and the urban poor (Hawkins et al., 2013; WHO/UNICEF Joint Monitoring Programme for Water Supply, 2017). Addressing marginalization and disparities in access to services will be important considerations to meet the SDGs.

Addressing the water and sanitation needs of people living in urban informal settlements will be essential for delivering on the inclusive promise of the SDGs. We examine the drivers and barriers that influence water and sanitation policy development, adoption, and implementation in urban informal settlements, focusing on low- and middle-income countries (LMICs) as defined by the World Bank (World Bank, 2019). We then consider the role that donor agencies and researchers can play in contributing to these aspects of the policymaking process, with a focus on our field of public health research.

The paper is structured as follows. We first establish context for the discussion by defining informal settlements and slums and discussing WASH conditions in these settings (Section 2). In Section 3, we describe the methods used for our literature review, including search strategy, inclusion/exclusion criteria, and data synthesis. We present our findings in Section 4, following the structure of drivers and barriers established in Section 3. Section 5 is a discussion of our findings, including key lessons, recommendations, and limitations of our review. Section 6 concludes the paper with directions for future work and policy implications.

## 2 Context of the discussion

### 2.1 What is an informal settlement?

The UN Human Settlements Programme (UN-Habitat) defines informal settlements as:

“residential areas where 1) inhabitants have no security of tenure vis-à-vis the land or dwellings they inhabit, with modalities ranging from squatting to informal rental housing, 2) the neighborhoods usually lack, or are cut off from, basic services and city infrastructure, and 3) the housing may not comply with current planning and building regulations, and is often situated in geographically and environmentally hazardous areas” (UN-Habitat, 2015).

UN-Habitat goes on to describe slums as “the most deprived and excluded form of informal settlements” (UN-Habitat, 2015). Slum households are defined as those “in which the inhabitants suffer one or more of the following ‘household deprivations': lack of access to improved water source, lack of access to improved sanitation facilities, lack of sufficient living area, lack of housing durability and lack of security of tenure” (UN-Habitat, 2016). However, terms such as informal settlement, slum, squatter settlement, and peri-urban area are often, but not always, used interchangeably (UN-Habitat, 2003). In this study, urban informal settlement and slum will be treated as synonyms.

Slums in urban and peri-urban areas present a significant and growing challenge with increasing urbanization. UN-Habitat estimates that, since 2000, the global slum population has grown by an average of 6 million people a year, with 90% of urban growth occurring in developing countries (UN-Habitat, 2016). In urban areas globally, approximately one-quarter of the population lives in informal settlements (United Nations, 2018). In sub-Saharan Africa, nearly 60% of people living in cities reside in slums (UN-Habitat, 2016). Compared to rural or non-slum urban populations, studies have found higher mortality and morbidity in slum settings, particularly among infants and children (Mberu et al., 2016; Garenne, 2010; Ezeh et al., 2017). The physical environment is an important reason for this higher burden: crowding and limited access to safe water and sanitation facilitate the spread of infectious diseases, including enteric infections (Hawkins et al., 2013).

### 2.2 Water and sanitation in informal settlements

Informal settlements and slums, by definition, often lack adequate water and sanitation infrastructure (UN-Habitat, 2015; UN-Habitat, 2016). Where water infrastructure exists, access may be uneven and availability may be intermittent (Pierce, 2017). Intermittent water availability has been associated with lower microbial quality and a higher risk of waterborne disease; it also forces households to store water, which can introduce contamination (Bivins et al., 2017; Kumpel and Nelson, 2016). In these cases, data suggests that residents typically rely on a combination of market solutions and community governance to fulfill their basic water and sanitation needs (Dos Santos et al., 2017; Cross and Morel, 2005). These small-scale providers are generally unregulated and their operations may be illegal (Subbaraman et al., 2013; Dagdeviren and Robertston, 2009). The resulting access may not be adequately safe, reliable, affordable, or dignified (Hutton and Chase, 2016; Saravanan et al., 2016).

Many residents of informal settlements rely on shared infrastructure, although this term encompasses a range of conditions. The number of households (and individuals) using a block of toilets, a single pit latrine, or a standpipe can vary widely, with important consequences (Corburn and Karanja, 2016). Shared sanitation has been linked to adverse health outcomes compared to individual household latrines (Fuller et al., 2014; Heijnen et al., 2014). Defined as any sanitation facility shared by more than one household, shared sanitation may also present security risks and cause stress and anxiety among women and girls, especially when shared among many people (Kwiringira et al., 2014; Sommer et al., 2015; Water and Sanitation for the Urban Poor (WSUP), 2018). The cost of shared facilities may be prohibitive for some households, and inadequate cleanliness, security, or privacy may discourage use (Corburn and Karanja, 2016, Mcgranahan, 2015).

Where sanitation facilities exist, excreta may not be properly managed by separating it from human contact and treating it in a suitable facility. In a study of 12 low- and middle-income cities, 98% of households used toilets, but only 29% of fecal waste was safely managed (Blackett et al., 2014; Hutton and Chase, 2016). Pit latrines are perhaps the most common form of sanitation in informal settlements, but space limitations, among other factors, create barriers to latrine replacement as well as to safe and hygienic pit-emptying (Chipeta et al., 2017; Jenkins et al., 2015). Unsafe effluent discharge or pit emptying may pollute the environment, spreading contamination to households and communities far beyond the source.

## 3 Methods

Given the breadth of the topic area and the diversity of available literature, a systematic review was not undertaken. Systematic reviews are most appropriate for a narrowly defined question, typically involving quantitative data (Collins and Fauser, 2005; Buckley et al., 2013; Ferrari, 2015). Despite its limitations, a narrative review format was determined to serve our purposes by permitting a broader research question that would allow us to capture a diversity of policy drivers and barriers from a wide range of literature sources (Ferrari, 2015; Grant and Booth, 2009).

The review draws on published literature identified through a structured search process of PubMed and Scopus using terms related to water and sanitation, including: policy*, decision*, framework, driver, barrier, challenge, scaling up, scale-up, implementation, adoption. Searches were also conducted using the above terms in combination with terms including slum, informal settlement, urban poor, low-income urban, and unplanned. Given the policy orientation, unpublished and grey literature was sought through searches of the New York Academy of Medicine Grey Literature Report, http://www.greylit.org/, websites of key stakeholders (the World Bank, the Water and Sanitation Program, WHO, UN-Habitat, Water and Sanitation for the Urban Poor, U.S. Agency for International Development, IRC), and basic search engines (google.com). Additional literature, both published and unpublished, was identified through snowballing and from the reference lists of key articles.

The review includes published and grey literature from January 1, 2000 to April 1, 2018; earlier publications were excluded as being less relevant to current trends in decision-making (e.g., the signing in September 2000 of the UN Millennium Declaration, from which the MDGs are derived). Although no geographic restrictions were placed on the searches, the literature was reviewed for relevance to LMIC urban settings. No language restrictions were placed on the search, but only English language documents or abstracts were reviewed. Where possible, we have drawn on reviews and documents that synthesize the available evidence in order to provide a more global picture of water and sanitation in informal settlements. As reflected in our search terms, we were most interested in literature related to policy adoption and implementation; however, we reviewed literature related to any aspect of the policymaking process.

We applied a typology developed by Pierce, which categorizes barriers to the provision of basic services in slums (Pierce, 2017). Pierce's typology includes five categories of barriers: economic, spatial, social, institutional, and political. A sixth barrier (informational) is added here. While Pierce solely focuses on barriers to service provision, we organized evidence of drivers as well, and we applied this classification to multiple aspects of policymaking. Within a given “type,” drivers and barriers, in some cases, may be complementary (e.g., availability of funding may be a driver, while lack of funding may be a barrier). In other cases, the distinction is more complex (e.g., collective action may drive policymaking, but an absence of collective action does not create a barrier to policymaking). The nature and relative importance of these factors also may be context-specific, depending on the relevant political economy. An exhaustive review and discussion of context, however, is beyond the scope of this paper.

## 4 Findings

### 4.1 Drivers

#### 4.1.1 Economic factors

The development, adoption, and implementation of WASH policies for informal settlements requires appropriate financing mechanisms, which can come through several channels. First, governments with national urban development policies are more likely to allocate financial resources to upgrading and improving informal settlements (World Bank Group, 2015b). Similarly, responsive government policies often include funding subsidies to support households that are otherwise unable or unwilling to pay for WASH utilities (World Bank, 2014; World Bank Group, 2015b). Governments can also facilitate private funding by offering incentives for investment in infrastructure projects (UN-Habitat, 2015). In some cases, microfinance programs can be an additional component of a broader policy solution to improve access to adequate housing (UN-Habitat, 2015). Finally, donor institutions can be an important economic driver: Dagdeviren and Robertson assert that the World Bank's focus on slum upgrading in the 1970s and 1980s arose because it was less expensive than alternative policy choices (Dagdeviren and Robertston, 2009).

#### 4.1.2 Social factors

Mobilized collective action can effect important changes in decision-making at higher levels (e.g., for the subsidized provision of services or the enforcement of sanitation regulations) (Mcgranahan, 2015; Dagdeviren and Robertston, 2009). In some cases, community organizing around water or sanitation has resulted in government recognition or improved land tenure, which are themselves barriers to safe WASH services (Mcgranahan and Mitlin, 2016). In others, collaboration among civil society and non-governmental organizations (NGOs) has resulted in improved provision of water and sanitation services for the urban poor (Overseas Development Institute, 2017c; Chaplin, 2011). In a review of 50 water supply and sanitation case studies, community participation and ownership were found to be among the most important factors in driving the successful provision of services to the poor (Murungi and Blokland, 2016).

Community participation can lead not only to increased government service provision but also to improved quality and sustainability of water and sanitation infrastructure. Community-led processes to implement WASH policies (e.g., to design and build community toilets) can increase the likelihood that the WASH infrastructure will align with community needs and priorities (Burra et al., 2003). A lack of available, accessible, and appropriate WASH facilities places a disproportionate burden on women, children, and the disabled; involving these groups as stakeholders in the design and planning process is therefore particularly important (Burra et al., 2003; Ganesh et al., 2019). Involving residents throughout the implementation process can also promote a sense of ownership that contributes to improved management and sustainability of infrastructure (Burra et al., 2003; Sanitation and Hygiene Applied Research for Equity (SHARE), 2014; World Bank Group, 2015a).

#### 4.1.3 Institutional factors

International institutions set global agendas (e.g., the United Nations 2030 Agenda, which includes the SDGs), which may influence governments to make decisions around policies and investments (Cronin et al., 2015). Many donors similarly prioritize their funding based on these international agendas (Clark and Gundry, 2004). The reliance on donor funding in many LMICs means that donor priorities, and the global trends they reflect, can have an important influence on decisions about how to invest in water and sanitation. Donor agencies, in addition to providing funding, can also push for increased accountability and provide technical support and capacity building to promote sustainability of WASH policies (Overseas Development Institute, 2017a, O'meally, 2013).

#### 4.1.4 Political factors

WASH policy and investment decisions largely depend on the government's overall policy regarding informal settlements. The three primary strategies for addressing slums are: (1) clearance through forced or legal evictions; (2) benign neglect; and (3) regularization of settlement conditions (e.g., upgrading) (Dagdeviren and Robertston, 2009; Satterthwaite et al., 2018). Upgrading policies for informal settlements often include the provision of WASH infrastructure and regularization of informal settlements (UN-Habitat, 2015). Where a political “champion” exists to push these policies forward, they will be more likely to achieve success (Overseas Development Institute, 2017a). Finally, good governance, including coordination across ministries and between national and sub-national offices, can contribute to the success of these policies (Overseas Development Institute, 2017b).

As discussed in relation to social factors (Section 4.1.2), civil society can influence these policies and the broader political economy of decision-making around water and sanitation services. Through voting and political representation, some residents of informal settlements have been able to persuade politicians to prioritize service provision to slums (Chaplin, 2011, Mcfarlane, 2008). This can create a strong political interest in providing and maintaining water and sanitation services despite high transaction costs (Dagdeviren and Robertson, 2016). In particular, when they have capacities and can build coalitions or networks, these groups can help increase accountability among policymakers and service providers (O'meally, 2013; World Bank, 2003). Increased social accountability can increase officials' likelihood of cooperating (Davis, 2004). However, these social accountability processes are context-specific and may be tenuous and difficult to scale (Chaplin, 2011, O'meally, 2013).

### 4.2 Barriers

#### 4.2.1 Economic factors

Economic and financial considerations have clear importance for policymaking. In particular, the cost of interventions is “an often-cited constraint for an investment decision, whether governments, the private sector, or households and individuals” (Hutton and Chase, 2016). Connecting informal settlements to water supply or sewerage may have high upfront costs due to their peripheral location or physical conditions (Dos Santos et al., 2017). In many countries, a lack of effective taxation systems may limit the funding available for infrastructure investments (UN-Habitat, 2015). Where funding is available, governments have been less willing to invest in infrastructure for sanitation than for water (Isunju et al., 2011; Cairncross et al., 2010; Chaplin, 2011).

One barrier to government investment may be fears around cost recovery, specifically the perception that residents of informal settlements may be unwilling or unable to pay enough to cover the investment and maintenance costs of services (Cross and Morel, 2005; Dos Santos et al., 2017; Paterson et al., 2007; Pierce, 2017). Numerous studies, however, have demonstrated willingness to pay for water services (Subbaraman et al., 2013). Indeed, individuals and households in informal settlements often pay higher rates for water services than residents of developed parts of the city (Dos Santos et al., 2017; Pierce, 2017). Willingness to pay for sanitation may be lower and may depend on factors such as the type of sanitation facility and the resident's tenure or ownership status (Isunju et al., 2011; Mcgranahan and Mitlin, 2016; Simiyu et al., 2017). Even when governments establish subsidies to improve affordability for the poor, these might also be captured by wealthier urban residents with existing service connections or resources to navigate the system (Cross and Morel, 2005; Mara et al., 2010, Wateraid, 2008). Therefore, cost and affordability constitute “only one dimension of access,” but can be an important factor throughout the policymaking process (Dos Santos et al., 2017).

#### 4.2.2 Spatial factors

A key spatial factor with regard to the development, adoption, and implementation of WASH policy is the location of the settlement. Informal settlements may be located on the periphery of a city or beyond its formal boundaries. Extending service infrastructure to these areas can be expensive or technically difficult; utilities may lack the necessary equipment or technical capacity or may be disinclined to allocate these resources for informal settlements, especially in light of concerns about residents' ability to pay (Dos Santos et al., 2017). In addition to increasing the volume of water required, connecting informal settlements to the water supply may shift the geography of demand away from established distribution centers and can affect the quality and regularity of the supply (Dos Santos et al., 2017; Pierce, 2017). Finally, if the settlement crosses administrative boundaries, then responsibilities for service provision may be unclear (Pierce, 2017; UN-Habitat, 2015).

As unplanned and often illegal communities, informal settlements tend to develop on land that is otherwise undesirable, including areas that are prone to flooding or landslides (Dagdeviren and Robertston, 2009; Pierce, 2017). Using spatial analysis, Olthuis et al. have shown that slums will even grow onto adjacent water bodies, further exacerbating residents' vulnerability to flooding and other disasters (Olthuis et al., 2015). A high water table or unstable ground make installing some types of infrastructure, such as pit latrines or septic tanks for flush toilets, problematic or even impossible.

The built environment poses similar technical challenges. Both density and housing quality are included in UN-Habitat's definition of a slum, and each characteristic complicates the provision of water and sanitation in these areas (Cross and Morel, 2005). Where land is scarce and population density is high, providing on-site sanitation at a household level or replacing pit latrines may not be possible. Narrow streets can make it difficult for utilities and service providers to access infrastructure for repairs or maintenance, including pit emptying (Hawkins et al., 2013). Finally, the low quality and limited durability of construction materials in informal settlements may not support the installation of permanent infrastructure like pipes or taps (Dagdeviren and Robertston, 2009). All of these challenges may act as disincentives to developing and adopting new WASH policies or implementing existing policies.

#### 4.2.3 Social factors

While residents' collective action, civic participation, and leadership can be drivers of policy development, adoption, and implementation; other social factors can act as barriers. For example, due to patterns of rural-urban migration, many residents of informal settlements may be unfamiliar with urban administrative units and agencies (UN-Habitat, 2015; World Bank Group, 2015b). In some cases, they may speak a different language than the one used by government officials (UN-Habitat, 2015; World Bank Group, 2015b). Even longtime residents of informal settlements may not have the time, resources, knowledge, or sufficient trust in local systems to engage in advocacy for themselves and their communities, to demand new policies or to hold officials accountable for policy implementation (World Bank Group, 2015b; De and Nag, 2016).

The ability of local groups to organize and advocate for themselves also depends on the social capital present in the community, as well as the power of the individuals. People who reside in informal settlements often experience marginalization through multiple pathways, including poverty, which can limit their ability to advocate for themselves. Informal settlements, in particular, often develop or are maintained as a result of discrimination against certain groups based on caste, ethnicity, race, or religion (Pierce, 2017). In many countries, these settlement patterns, along with underinvestment in some communities, are a legacy of historical and/or colonial systems of power and division (Saravanan et al., 2016; Corburn and Karanja, 2016; Chaplin, 2011; Appelblad Fredby and Nilsson, 2013; Dill and Crow, 2014; Fox, 2014). These same social factors of discrimination and exclusion may lead to these groups being treated unequally under government policies and to uneven policy implementation (De and Nag, 2016).

In some places, a lack of strong government control has allowed criminal networks to thrive within informal settlements, controlling the water supply and demanding high prices (Gandy, 2008). These networks may charge fees to access public water supplies or to collect water from tankers, taking particular advantage of situations of water shortages (Gandy, 2008; Truelove, 2011; Tutu and Stoler, 2016). They may even pirate public water supplies for the production of sachet water that is then sold to residents of informal settlements (Tutu and Stoler, 2016). These networks may be acting in collusion with government officials or may be beyond the state's control (Gandy, 2008).

#### 4.2.4 Institutional factors

Informal settlements face a number of institutional barriers to the development and adoption of WASH policies, including a lack of clear institutional mandates and policy coordination between government agencies as well as among donor institutions (Overseas Development Institute, 2017b). Related to this is a lack of an institutional “planning culture” in which stakeholder agencies lack the capacity, time and/or resources for appropriate urban planning and decision-making (Ramôa et al., 2017). Another common barrier relates to tenure status: some countries, like India, have defined multi-tiered systems of official recognition of tenure status; in other places, the distinctions are less well defined (Subbaraman et al., 2012). Indeed, governments are at times unwilling to extend services to informal settlements because they fear that doing so would imply recognition (Dagdeviren and Robertston, 2009). If the settlement is on private land, the government would have to purchase the land or seek permission from the owner before constructing new infrastructure; many owners refuse because they do not want the settlement to be permanent (Tshishonga and Mafema, 2011). Without official government recognition or permission, utilities may not be allowed to provide services to these areas, or they may simply not be obligated (Mcgranahan, 2015). Thus, issues of land tenure and recognition may preclude the development of WASH policies for informal settlements (Subbaraman et al., 2013; Saravanan et al., 2016).

Legal frameworks, particularly sanitation regulatory frameworks, are a necessary tool for ensuring more inclusive service provision (Kerstens et al., 2016). At the same time, laws can also foster greater exclusion and deprivation, especially when they are outdated or require inappropriate technical solutions (Cairncross et al., 2010). If the buildings themselves do not meet certain legal standards, they may be exempted from formal service provision (Dagdeviren and Robertston, 2009). In addition, small-scale providers, a key source of water and sanitation services in slum communities, may be threatened by regulations. In many settings, they operate in an uncertain space “between official tolerance and illegality” (Dagdeviren and Robertston, 2009). Confusing or mismatched laws, especially in the absence of a broader policy vision, can thus be a barrier to the implementation of inclusive WASH policies, even when such policies exist.

#### 4.2.5 Political factors

In addition to institutional factors, a number of political factors can present barriers to implementation of WASH policies for informal settlements, including corruption and patronage. Research from South Asia suggests that corruption is widespread throughout water and sanitation service providers (Asthana, 2008; Davis, 2004; Gandy, 2008). This corruption can take a variety of forms, from small bribes for household-level repairs to price-fixing and delayed construction of system-level water infrastructure (Gandy, 2008). Without strong institutional leadership, individual leaders are likely to prioritize personal and political interests and allegiances, which may actively conflict with existing policies and the needs of informal settlements (Overseas Development Institute, 2017b; Fox, 2014; Chaplin, 2011). Indeed, implementing policies to provide services to informal settlements requires a commitment of resources, which may be unpopular with other urban residents, especially among the middle and upper class (Dagdeviren and Robertston, 2009; Fox, 2014; Gandy, 2008). In other cases, politicians may seek to gain local support by securing access to services like water and sanitation, but maintenance can be a problem (Mcgranahan, 2015).

Another barrier is the fragmentation of responsibility around water and sanitation (Cross and Morel, 2005; Dagdeviren and Robertston, 2009; Saravanan et al., 2016; Cronin et al., 2015). Multiple sectors have a stake in water and sanitation policy decisions, and, in many countries or cities, no single entity has the authority to take action. Decentralization has been promoted by international aid agencies to increase efficiency and accountability in service provision (Asthana, 2008; Wolf, 2007). As a result, local governments have been given responsibility for delivering water and sanitation services but often lack the necessary funding or capacity (Cairncross et al., 2010; Wolf, 2007). These barriers are not limited to informal settlements, but responsibility in such settings tends to be even more diffused. Depending on the community, the provision of basic services often involves some combination of public, private, and voluntary sector entities (Narayanan et al., 2017). The landscape of decision-making around policy implementation is therefore more complicated.

Even within the same city, two informal settlements may differ greatly in terms of demographics, history, legal standing, and power structures (Swyngedouw et al., 2002). Different political interests may be at play locally. The differences both shape and reflect the governance structure and political economy of each informal settlement. The ability of residents to advocate for resources and specific investments is similarly varied (Hawkins et al., 2013; Chaplin, 2011). Understanding these dynamics, and where the power resides in any particular informal settlement, is important for understanding barriers to the development and implementation of effective water and sanitation policies (Hawkins et al., 2013; Swyngedouw et al., 2002).

#### 4.2.6 Informational factors

A lack of data for decision-making is an important barrier to WASH policy development and implementation, and the lack of information on informal settlements often stems from other barriers. Informal settlements' existence at the physical and legal edges of society can make them easy to overlook, by accident or intention. As described above, some governments do not recognize informal settlements and thus do not include them in official data collection processes (UN-Habitat, 2015). Real estate and administrative records can be an important source of population information; lack of tenure status or rapid (and informal) development in slum areas may leave residents underrepresented in such records (Cross and Morel, 2005). Without accurate land surveys and other data, policymakers cannot identify needs, develop policy responses, monitor implementation performance, or provide appropriate oversight (Overseas Development Institute, 2017b; UN-Habitat, 2015).

In many cases, national surveys and censuses undercount or exclude marginal populations, including informal settlements (Bartram et al., 2014; Lucci et al., 2018). This occurs for a variety of reasons: certain areas or individuals may be considered “hostile and unsafe or hard-to-reach,” including places where the environment is unsanitary (Lucci et al., 2018). Political and social factors may also drive decisions around sampling frames; political incentives, in particular, may “render populations in slum settlements invisible in the data” (Lucci et al., 2018). Even when they are included, the sampling methodology of surveys often does not permit disaggregation by subgroups (Hutton and Chase, 2016). This undercounting can have important implications for the investments made in these communities.

Often, data collection is guided by indicators such as those established by the MDGs and SDGs. However, UN-Habitat cautions that these indicators are tools for obtaining consensus and direction; that is, they are political, not merely technical (UN-Habitat, 2003). A number of papers suggest that the indicators and benchmarks in use are not appropriate for slum settings (Murungi and Blokland, 2016; Satterthwaite, 2016; Lucci et al., 2018). The type of measures used may not capture the situation in slums. The authors argue that, even if such targets are achieved, they will not adequately address health risks and other dimensions of safe access such as affordability.

Finally, very limited evidence exists about which WASH interventions work in informal settlements and under what conditions (Hutton and Chase, 2016; Dos Santos et al., 2017; Brown et al., 2015; Turley et al., 2013; Lilford et al., 2017). Much of the research has focused on the more visible water and sanitation gap in rural areas (Dos Santos et al., 2017; Ezeh et al., 2017). However, findings from studies in rural communities may not be generalizable to urban settings, and they may be especially ill suited to address the specific challenges of informal settlements. The focus on pit latrines as a solution is an example of this mismatch (Satterthwaite, 2016). Thus, decision-makers often lack appropriate evidence to guide policy development related to informal settlements.

## 5 Discussion

We have outlined drivers and barriers to WASH policy development, adoption, and implementation in informal settlements in LMICs. Key drivers and barriers are summarized in Table 1 (by type). In reviewing the literature, we identified far fewer drivers than barriers. Few documented examples exist of communities, governments, and donor institutions coming together to develop, adopt and implement WASH policies for informal settlements. Instead, a preponderance of the literature focuses on settings where a range of barriers has prevented this from happening. It is not clear whether this disproportionate volume of evidence on barriers is an accurate reflection of realities on the ground, of researchers' interests, or both.

**Table 1:** Summary of key drivers and barriers of WASH policy development, adoption, and implementation for informal settlements in LMICs by type.

Type	Drivers	Barriers
Economic	• Appropriate financing mechanisms through government policy, donor institutions, and private sector infrastructure investments	• Insufficient funding for infrastructure investments • Lack of cost recovery mechanisms to cover high upfront costs and maintenance, especially for sanitation
Spatial		• Geographic characteristics of informal settlements (e.g. peripheral location, on unstable land or areas prone to flooding) • High housing density and poor construction of settlements
Social	• Community mobilization and collective action for government service provision and regularization of informal settlements	• Lack of resources (e.g. literacy, language skills, time) • Lack of social capital and social cohesion • Social characteristics of informal settlements (e.g. crime) • Marginalization and discrimination against residents of informal settlements
Institutional	• Global agendas and donor prioritization of inclusive WASH policies and social accountability	• Lack of clear mandates, policy coordination, and legal/planning frameworks • Insufficient capacity, time and/or resources for urban planning and policymaking • Lack of tenure status of residents • Barriers to official recognition of tenure
Political	• Political support for good governance and urban development policies • Citizen participation and civil society mobilization for inclusive WASH policies and social accountability	• Corruption, patronage • Decentralization and fragmentation of responsibility for WASH service provision • Lack of political will to meet the needs of residents of informal settlements
Informational		• Lack of appropriate global indicators for informal settlements • Lack of accurate, representative, and relevant data on informal settlements, with sufficient sample sizes for disaggregation • Insufficient evidence of “what works” in informal settlements

While estimates suggest that cities overall have high rates of coverage of improved WASH facilities, disparities remain between the urban rich and urban poor, as well as between different neighborhoods. Informal settlements, in particular, have relatively poor WASH conditions. It is easy to blame these conditions on economic barriers, but the reality is more complex. Spatial barriers, arising from the location and physical conditions of informal settlements, can limit the available options for the development of WASH policy responses. In addition, arguably the most important factor determining WASH access in informal settlements is the establishment of tenure status in the community. Institutional and political barriers may prevent recognition of tenure status, which is often required for service provision. Finally, a lack of data on the WASH situation in informal settlements, combined with the marginalization of residents due to poverty and other social factors, can make it difficult to influence decision-makers to develop, adopt and implement appropriate WASH policy solutions.

From the literature reviewed on drivers of WASH policy in informal settlements, some evidence emerges of what works. First, urban development policies can reduce the existence of informal settlements in general and can lay the groundwork for more equitable service provision across urban populations, especially when backed by financial and technical support from donor institutions. Community mobilization, including in collaboration with NGOs, can create political pressure for regularization and upgrading of informal settlements. Finally, coalitions of residents of informal settlements can increase social accountability of political leaders through voting and advocacy.

Researchers and donor agencies may be able to contribute to supporting drivers and addressing barriers in several ways. Donors can promote responsive policies and prioritize WASH service provision for informal settlements as a development and economic issue. Donors and researchers can disseminate and build local capacity to use existing planning frameworks and process guides (Kerstens et al., 2016; Ramôa et al., 2017). In some cases, especially when existing frameworks may not be appropriate to local contexts, researchers may help to model options and then work with decision-makers to identify locally appropriate interventions (Mills et al., 2018). They can also contribute to coalitions of civil society, NGOs, and donor institutions working within the WASH sector as well as those working on broader agendas (e.g., tenure security, health, and human rights) (Overseas Development Institute, 2017a). These and other stakeholders can benefit from the data collection, analysis, and visualization expertise of researchers. Policy analysis and implementation research, with careful analysis of the drivers of success, can be particularly useful (Overseas Development Institute, 2017a). While results may not be generalizable to all contexts, learning “what works” may provide evidence to inform policymakers in similar settings. This is in line with SDG 6. A, “expand international cooperation and capacity-building support to developing countries in water- and sanitation-related activities and programmes” (UN, 2017).

For public health researchers in particular, while disagreements exist about how much health concerns drive investment in water and sanitation, it is important to recognize that “purely evidence-based policymaking is unrealistic and naïve” (Wsp, 2011). Still, working in collaboration with experts from other fields and with individuals and organizations who are knowledgeable about the local context, researchers can leverage the discipline's strengths and governments' interest in population-level health outcomes to support advocacy efforts. Specifically, public health researchers can make important contributions in three key areas: (1) identifying appropriate health outcome indicators; (2) ensuring representative data that can be disaggregated by sub-group; and (3) carrying out impact evaluations of water and sanitation interventions in urban informal settlements. While evidence may not be the only driver of policy decisions, filling these data gaps can reduce an important barrier to WASH policy development.

### 5.1 Limitations

While this review has sought to present evidence from a wide range of contexts, it was not a systematic review and does not purport to be exhaustive. Drawing on reviews that synthesized evidence from a range of contexts (where possible) should improve the generalizability of the results. Selection bias remains a concern. Another key limitation is that, due to the nature of the available literature, this review focuses on drivers and barriers as identified by the authors. Very few studies have attempted to directly study the drivers and barriers of any aspect of the WASH policymaking process in informal settlements or slums. The evidence overall is weak, coming primarily from case studies or cross-sectional surveys.

The search strategy did not include a comprehensive list of terms used for informal settlements in specific contexts, as has been done in recent reviews that included terms such as “ghetto” and “shanty town” in their search terms (Ezeh et al., 2017; Lilford et al., 2017). Instead, our search strategy was limited to the terminology typically used by the UN and research studies. It is possible that focusing the search on water and sanitation failed to retrieve relevant literature, as these issues can also be discussed under the heading of basic service provision or slum upgrading. Identifying additional sources through snowballing and from reference lists lessens this concern.

Finally, while this review has treated water and sanitation together, a combined approach may not be appropriate in every context (Murungi and Blokland, 2016). Policymaking processes and delivery systems may be distinct. Identifying a set of shared drivers and barriers may be too crude, burying important nuances. Progress in sanitation has lagged behind water worldwide, especially in urban areas. It may be valuable to consider water and sanitation separately, to tease out the separate challenges that have hampered the expansion of sanitation in dense urban environments.

## 6 Conclusion

Despite global progress, informal settlements continue to have lower coverage of improved WASH facilities than other urban areas. Addressing disparities in access in these settings will require a coordinated, multi-sectoral effort that includes both top-down and bottom-up approaches to support drivers and reduce barriers to inclusive WASH policymaking. Top-down approaches can take the form of donor agendas and government urban development policies, while bottom-up approaches would involve community mobilization and advocacy. Researchers can play a role in capacity development by providing additional data collection, analysis, and visualization expertise. Public health researchers, in particular, can leverage the importance of health as a development issue and build the evidence case on health outcomes related to water and sanitation in urban informal settlements. Inter-disciplinary collaborations among researchers, governments, civil society, private sector, and donors may allow for a better understanding of the context-specific drivers and barriers to decision-making and lead to the identification of sustainable policy solutions for improved WASH services in urban informal settlements.
